# Testing the psychometric properties of the Environmental Attitudes Inventory on undergraduate students in the Arab context: A test-retest approach

**DOI:** 10.1371/journal.pone.0195250

**Published:** 2018-05-14

**Authors:** Entesar Ali AlMenhali, Khalizani Khalid, Shilpa Iyanna

**Affiliations:** 1 College of Business Administration, Abu Dhabi University, Abu Dhabi, UAE; 2 Department of Management, College of Business Administration, Abu Dhabi University, Abu Dhabi, UAE; 3 Department of Marketing, College of Business Administration, Abu Dhabi University, Abu Dhabi, UAE; Aligarh Muslim University, INDIA

## Abstract

The Environmental Attitudes Inventory (EAI) was developed to evaluate the multidimensional nature of environmental attitudes; however, it is based on a dataset from outside the Arab context. This study reinvestigated the construct validity of the EAI with a new dataset and confirmed the feasibility of applying it in the Arab context. One hundred and forty-eight subjects in Study 1 and 130 in Study 2 provided valid responses. An exploratory factor analysis (EFA) was used to extract a new factor structure in Study 1, and confirmatory factor analysis (CFA) was performed in Study 2. Both studies generated a seven-factor model, and the model fit was discussed for both the studies. Study 2 exhibited satisfactory model fit indices compared to Study 1. Factor loading values of a few items in Study 1 affected the reliability values and average variance extracted values, which demonstrated low discriminant validity. Based on the results of the EFA and CFA, this study showed sufficient model fit and suggested the feasibility of applying the EAI in the Arab context with a good construct validity and internal consistency.

## Introduction

In environmental psychology studies, several models on the substantial influence of human behaviors on the natural world have included environmental attitudes as an indicator of behavior that either improves or worsens the environmental quality. Attitude is defined as an enduring evaluation of people, objects, or ideas [1], a combination of motivational, emotional, perceptual, and cognitive processes with respect to some aspect of the environment [2], and the learned predisposition to consistently respond to a given object [3]. Environmental attitudes are more of an individual concern for the physical environment, which is related to the degree of cognitive, affective, and behavioral concerns toward the environmental problems [4].

Environmental attitudes have been considered a relevant part of environmental psychology models and therefore, are often included in measures of environmental behavior, sometimes considering school students [5–7], university students [8–10], and the general community together [11–13].

Studies on environmental attitudes heavily rely on the subjective measures of environmental cognitions and environmental affection in comparison to objective measures [14–16]. Environmental attitudes are a latent construct and thus unlikely to be observed directly [15]. Traditionally, attitude measurement is designed as direct self-report methods [17–18]. Conventionally, environmental measurement is designed as self-administered methods [19–20] based on the perceived ecological issues and problems. Although some studies have measured environmental attitudes, only two measurements have received much attention due to their validity and reliability assessed [21–22] across cultures.

To avoid the dated issues of the general environmental topics, Dunlap and Van Liere [23] developed the new ecological paradigm (NEP) scale to measure the overall relationship between humans and the environment. Later, the revised version of the NEP (NEP-R) [24] was developed as a better alternative with stronger psychometric properties using sound, grounded theories. Over 49 studies worldwide have used this scale to examine and better comprehend the environmental attitudes driven by values and beliefs about the natural environment and its management. The NEP scales measure ecocentric systems of beliefs as opposed to the anthropocentric systems of beliefs [21]. The 15 items tabulated in the five-factor model of the NEP-R is the most widely-used measure to investigate environmental attitudes. Dunlap, Van Liere, Mertig, and Jones [24] presented a five-factor model that emphasized the awareness of consequences to reflect environmental concerns and values to explain the underlying beliefs from a global perspective. The five-factor model, which encompasses balance of nature, eco-crisis, anti-exemptionalism, limits of growth, and human domination, consists of 15 items. This scale investigates multiple environmental phenomena related to beliefs, attitudes, intentions, and behaviors. It measures multiple environmental issues including pollution, waste management, recycling, and natural resources. Thus, this measure can be considered a multiple-topic assessment method [21].

Much attention was given by a previous study to treat the NEP-R as a measure of environmental beliefs rather than ecological worldview as proposed by Dunlap et al. [24]. Dunlap explains that environmental beliefs provide a more accurate interpretation due to the degree to which respondents view and perceive the world from an ecological viewpoint [21]. Turaga, Howarth, and Borsuk [25] posited that the importance of the NEP/NEP-R for measuring the environmental concerns in the world has been emphasized both from theoretical and practical viewpoints, and used successfully in cross-cultural research [21]. Recent studies have revealed that the NEP-R yields a 2-factor model [26], 4-factor model [27], and 5-factor model [28] instead of unidimensionality of the 15 items. Due to extensive use of the NEP-R for classifying people’s ecological worldview and monitoring changes in worldview over time, the NEP was problematic because of its overestimation [22, 28–32].

The NEP-R has been argued for being overly simplistic and outdated [33–34]. These studies have argued that the NEP-R scale has omitted critical components of the pro-ecological worldview, and the ecocentric perspective resulting from the late twentieth-century environmental ethics literature was excluded. The NEP-R has also been criticized for being a poor predictor of environmental behavior. Earlier studies showed that the relation between the NEP-R and attitude in predicting behavior is weak; thus, it failed to measure worldview precisely [22, 27, 35]. Psychometric analyses conducted on the 15 items showed that the NEP-R is best represented by its five correlated dimensions [28, 36]. These studies have shown that testing the unidimensionality of the NEP/R’s 15 items entails a statistical bias when used as a predictive score or outcome variable. A meta-analysis of 69 studies using the NEP/NEP-R showed that differences in sample type and scale length have a significant effect on NEP scores, making comparison of scores across studies difficult [37].

Milfont and Schultz (2016) have argued that environmental beliefs should be supported by environmental attitudes to define the environmental responsibilities that are reflected from the evaluation of environmental consequences. Bronfman et al. [38] posited that culture is the key source of social influence because of its proximity to, permanent contact with, and authority over environmental attitudes. The environmental attitudes inventory (EAI) [22] was established to address the limitations of the NEP-R. The EAI was developed as an alternative to the NEP and suggests a complex multidimensional structure of environmental attitudes to measure individual’s beliefs regarding the natural environment across 12 dimensions [22, 39]. The multifaceted structure of environmental attitudes was developed from a pool of 200 scale items drawn from established pro-environmental behavior measures: the NEP [21, 24], the Ecological WorldView Scale [40], and the Environmental Perception Scale [22, 41]. The psychometric criteria for the 200 items were tested unidimensionally and multidimensionally for reliability, validity, and intercorrelation evidence; they were revised as a short version of the 12-factor model with six items per scale [22].

Recent studies have shown that the EAI yields a 6-factor model [42], 9-factor model [39], 10-factor model, and 11-factor model [34], the one-dimensional environmental movement activism [35], an 11-factor model [22], 7-factor model [43], and a 6-factor model [44]. Even though the EAI is considered an appropriate tool to measure the influence of ecological initiatives, factors such as socio-demographic effects on cultural biases [45], social-desirability bias [46], and non-response bias [47] may affect the internal consistency of the data from a different population. Like other replication studies, the results of the psychometric analysis of the EAI results show that the extracted factor structures are distinct from the original constructs. It is argued that there is a possibility of the existing studies not being able to assemble enough evidence to draw a definite conclusion about the dimensionality of environmental attitudes in cross-cultural research [48–50]. Thus, it is argued that the generalization of environmental dimensions to underlie the evaluation of the ecological belief, values, and attitudes across cultures is not conclusive. More studies should be conducted to include other distinctive cultures when measuring environmental attitudes to capture the differences in perceived ecological responsibilities and ecological consequences.

Therefore, it is necessary to measure the level of ecological information available within a specific culture, as well as how people perceive ecological issues that shape their environmental attitudes. Thus, it is necessary to utilize a suitable instrument to carry out this function. In psychology-ecology research not many studies have been designed to establish what is essential in the adaptation process of a questionnaire in a culture-specific context [51–52]. Careful translation is not enough to validate a survey because linguistic terms must be relevant to the social and cultural conditions of the population studied [53]. Psychometric measures within a specific context are needed because each society has its beliefs, habits, and attitudes that reflect a country’s culture. López and Amerigo [54] concur that careful identification of individual norms and values for each ecological issue is required to establish meaningful results. A fundamental aspect of developing a useful and meaningful tool is the number of questions. Furthermore, the time taken to answer the questions is another essential factor in selecting the questionnaire to be used. This is consistent with recent concerns related to response burden or response fatigue due to a large number of questionnaire items that affect willingness to participate [47]. The use of a questionnaire to assess young adults’ environmental attitudes when facing ecological issues is an appropriate source for investigating attitudes that influence responsibilities toward ecology, based on the perceived consequences of ecological problems. Attitudes are a latent construct and thus must be inferred from overt responses [55–56]. The explicit measurement technique of a direct self-report method using a questionnaire is the most popular method for measuring environmental attitudes [21–22].

This study aimed to investigate the reliability, construct validity, and factor structure of Milfont and Duckitt’s EAI [22] owing to the multidimensional nature of environmental attitudes. The EAI assesses broad perceptions regarding ecology and the factors affecting its quality. Based on the arguments discussed, the 12-factor EAI seems to cover the most widely examined environmental attitudes factors sufficiently. The psychometric properties of the EAI were measured for two primary purposes: (1) to determine whether the 72-item EAI is suitable for use in the Arab context; and (2) to establish the baseline framework for environmental attitudes.

## Study 1

### Materials and methods

Hagel (2014) advocates that a psychometric theory requires unidimensionality, while Ziegler and Hagemann (2015) posited that this technique is parallel to testing the dimensionality of items. To determine which scale items of the EAI should represent a common, latent construct, an exploratory factor analysis (EFA) was employed. This varimax rotation method is employed to develop a smaller number of variables that will account for variance of the latent constructs. Varimax rotation is used to maximize the variance of the factor loadings on all constructs in a factor matrix. This technique has the effect of differentiating the original variables by extracted factors [57] due to factor intercorrelations. As a result, it reduces many items and latent constructs into fewer numbers of factors. This analysis is important to identify critical dimensions that would compromise the structure and criterion variables in subsequent analyses. Furthermore, it enables understanding of the structure of the variables and determination of the correlation among them in the data set [58]. In the second stage of the EAI testing, confirmatory factor analysis (CFA) is used to confirm the factor structure and specify how the construct is measured [57].(For this study, the analyses were based on an item pool of 72 items derived from Milfont and Duckitt’s model [22].

### Participants

An anonymous and consented questionnaire was administered to final year undergraduates at Abu Dhabi University, UAE, and 59.2% (148) of the students voluntarily agreed to participate. Of the subjects, 52.0% (130) eligible for the EAI measurement provided complete and consistent responses. Patterns of non-response were shown by 102 subjects (40.8%). Eighteen incomplete and inconsistent responses were identified, withdrawn, and not analyzed as part of the valid responses. A total of 130 participants (92 females and 38 males) with ages ranging from 21 to 35 years (M = 28, SD = 0.745) sharing very similar socio-demographic profiles (males and higher age group) from social sciences and science and technology colleges completed the questionnaire.

### Instruments

The twelve dimensions of the 72-item questionnaire developed by Milfont and Duckitt [22] were used to test the validity of the EAI in the Arab context. No ethical issues were present in this study as permission was sought from the original author to use the measure (Table 1 and Table 2). The study also was approved by the Institutional Review Board for Human Subjects Research (IRB) of Abu Dhabi University. Responses to all measures were made on a 7-point scale from 1 = strongly disagree to 7 = strongly agree. The following measures along with demographic information (e.g., age, gender, and program) were included in the questionnaire. None of the 72 items were translated into the Arabic language because English is the communication medium for teaching and learning, and all students are expected to meet the specified minimum score on the English proficiency of International English Language Testing System (IELTS) at the time of admission. The EAI consisted of 72 items that assess the 12 dimensions of environmental attitudes.

**Table 1:** The 12-factor model; 72 items of the environmental attitudes inventory scales [22].

*Scale 1*:*Enjoyment of nature (EN)* *The belief that enjoying time in nature is pleasant and preferable to spending time in urban areas versus the belief that enjoying time in nature is dull*, *boring*, *and not enjoyable and not preferable to spending time in urban areas*
I really like going on trips into the countryside, for example, to forests or fields^a^
I think spending time in nature is boring [r]^a^
Being out in nature is a great stress reducer for me^a^
I have a sense of well-being in the silence of nature
I find it more interesting in a shopping mall than out in the forest looking at trees and birds[r]
I find it very boring being out in wilderness areas[r]
*Scale 2*:*Support for interventionist conservation policies (SI)* *Support for conservation policies regulating industry and the use of raw materials*, *and subsidizing and supporting alternative eco-friendly energy sources and practices versus opposition to such measures and policies*.
Industries should be able to use raw materials rather than recycled ones if this leads to lower prices and costs, even if it means the raw materials will eventually be used up[r]^a^^b^
I am opposed to governments controlling and regulating the way raw materials are used to try and make them last longer[r]^a^^b^
People in developed societies are going to have to adopt a more conserving lifestyle in the future^a^^b^
I don’t think people in developed societies are going to have to adopt a more conserving lifestyle in the future[r]
Governments should control the rate at which raw materials are used to ensure that they last as long as possible
Controls should be placed on industry to protect the environment from pollution, even if it means things will cost more
*Scale 3*: *Environmental movement activism (EM)* *Personal readiness to actively support or get involved in organized action for environmental protection versus disinterest in or refusal to support or get involved in organized action for environmental protection*
I would NOT get involved in an environmentalist organization[r]^a^^b^
Environmental protection costs a lot of money. I am prepared to help out in a fund-raising effort^a^^b^
I would not want to donate money to support an environmentalist cause[r]^a^^b^
I would like to join and actively participate in an environmentalist group
I don’t think I would help to raise funds for environmental protection[r]
I would like to support an environmental organization
*Scale 4*: *Conservation motivated by anthropocentric concern (CM)* *Support for conservation policies and protection of the environment motivated by anthropocentric concern for human welfare and gratification versus support for such policies motivated by concern for nature and the environment as having value in and of themselves*
Conservation is important even if it lowers peoples’ standard of living[r]^a^^b^
We need to keep rivers and lakes clean to protect the environment, and NOT as places for people to enjoy water sports[r]^a^^b^
We should protect the environment even if it means peoples’ welfare will suffer[r]^a^^b^
One of the most important reasons to keep lakes and rivers clean is so that people have a place to enjoy water sports
Nature is important because of what it can contribute to the pleasure and welfare of humans
The thing that concerns me most about deforestation is that there will not be enough lumber for future generations
*Scale 5*: *Confidence in science and technology (CST)* *Belief that human ingenuity*, *especially science and technology*, *can and will solve all environmental current problems and avert or repair future damage or harm to the environment*, *versus belief that human ingenuity*, *especially science and technology*, *cannot solve all environmental problems*
Science and technology will eventually solve our problems with pollution, overpopulation, and diminishing resources^a^^b^
The belief that advances in science and technology can solve our environmental problems is completely wrong and misguided[r]^a^^b^
Modern science will solve our environmental problems^a^^b^
Modern science will NOT be able to solve our environmental problems[r]
We cannot keep counting on science and technology to solve our environmental problems[r]
Humans will eventually learn how to solve all environmental problems
*Scale 6*: *Environmental fragility (EF)* *Belief that environment is fragile and easily damaged by human activity*, *and that serious damage from human activity is occurring and could soon have catastrophic consequences for both nature and humans versus belief that nature and the environment are robust and not easily damaged in any irreparable manner*, *and that no damage from human activity that is serious or irreparable is occurring or is likely*
People who say that the unrelenting exploitation of nature has driven us to the brink of ecological collapse are wrong[r]^a^
Humans are severely abusing the environment^a^
The idea that the balance of nature is terribly delicate and easily upset is much too pessimistic[r]^a^
I do not believe that the environment has been severely abused by humans [r]
If things continue on their present course, we will soon experience a major ecological catastrophe
When humans interfere with nature, it often produces disastrous consequences
*Scale 7*: *Altering nature (AN)* *Belief that humans should and do have the right to change or alter nature and remake the environment as they wish to satisfy human goals and objectives versus belief that nature and the natural environment should be preserved in its original and pristine state and should not be altered in any way by human activity or intervention*
I’d prefer a garden that is wild and natural to a well-groomed and ordered one[r]^a^^b^
Human beings should not tamper with nature even when nature is uncomfortable and inconvenient for us[r]^a^^b^
Turning new unused land over to cultivation and agricultural development should be stopped[r]^a^
When nature is uncomfortable and inconvenient for humans, we have every right to change and remake it to suit ourselves^a^^b^
Grass and weeds growing between pavement stones really looks untidy
I’d much prefer a garden that is well-groomed and ordered to a wild and natural one
*Scale 8*: *Personal conservation behavior (PC)* *Taking care to conserve resources and protect the environment in personal everyday behavior*, *versus the lack of interest in or desire to take care of resources and conserve in one’s everyday behavior*
I am NOT the kind of person who makes efforts to conserve natural resources[r]^a^
Whenever possible, I try to save natural resources^a^
I always switch the light off when I don’t need it on any more^a^
In my daily life, I try to find ways to conserve water or power
I bothered to save water or other natural resources[r]
In my daily life, I’m just not interested in trying to conserve water and/or power[r]
*Scale 9*: *Human dominance over nature (HD)* *The belief that nature exists primarily for human use versus the belief that humans and nature have the same right*
Humans are no more important than any other species[r]^a^
Human beings were created or evolved to dominate the rest of nature
Plants and animals exist primarily to be used by humans
I DO NOT believe humans were created or evolved to dominate the rest of nature[r]
Plants and animals have as much right as humans to exist[r]
Humans were meant to rule over the rest of nature
*Scale 10*: *Human utilization of nature (HU)* *The belief that economic growth and development should have priority rather than environmental protection versus the belief that environmental protection should have priority rather than economic growth and development*
Protecting peoples’ jobs is more important than protecting the environment^a^^b^
Humans do NOT have the right to damage the environment just to get greater economic growth[r]^a^^b^
The benefits of modern consumer products are more important than the pollution that results from their production and use^a^^b^
Protecting the environment is more important than protecting economic growth[r]
Protecting the environment is more important than protecting peoples’ jobs[r]
The question of the environment is secondary to economic growth
*Scale 11*: *Eco-centric concern (EC)* *A nostalgic concern and sense of emotional loss over environmental damage and loss*, *versus the absence of any concern or regret over environmental damage*
I do not believe protecting the environment is an important issue[r]^a^^b^
Despite our special abilities, humans are still subject to the laws of nature^a^^b^
It does NOT make me sad to see natural environments destroyed[r]^a^^b^
It makes me sad to see forests cleared for agriculture
The idea that nature is valuable for its own sake is naïve and wrong[r]
Nature is valuable for its own sake
*Scale 12*: *Support for population growth policies (SP)* *Support for policies regulating population growth and concern about overpopulation versus lack of any support for such policies and concern*
Families should be encouraged to limit themselves to two children or less^a^
We should never put limits on the number of children a couple can have[r]^a^
We would be better off if we dramatically reduced the number of people on Earth^a^
The government has no right to require married couples to limit the number of children they can have[r]
A married couple should have as many children as they wish, as long as they can adequately provide for them[r]
Our government should educate people concerning the importance of having two children or less

**Notes:** All items were measured on a 7-point scale, where 1 = strongly disagree; 2 = disagree; 3 = somewhat disagree; 4 = neither agree nor disagree; 5 = somewhat agree; 6 = agree; 7 = strongly agree

^a^ denotes items retained in the 35-item shortened version of the inventory for Study 1

^b^ denotes items retained in the 21-item shortened version of the inventory for Study 2. Items followed by [r] were reverse coded for analysis

**Table 2:** The Cronbach’s alpha value for each dimension in the study by Milfont and Duckitt [22].

Scales	Cronbach’s alpha values
*Scale 1*:*Enjoyment of nature (EN)*	0.79
*Scale 2*:*Support for interventionist conservation policies (SI)*	0.88
*Scale 3*: *Environmental movement activism (EM)*	0.89
*Scale 4*: *Conservation motivated by anthropocentric concern (CM)*	0.62
*Scale 5*: *Confidence in science and technology (CST)*	0.73
*Scale 6*: *Environmental fragility (EF)*	0.84
*Scale 7*: *Altering nature (AN)*	0.89
*Scale 8*: *Personal conservation behavior (PC)*	0.90
*Scale 9*: *Human dominance over nature (HD)*	0.89
*Scale 10*: *Human utilization of nature (HU)*	0.84
*Scale 11*: *Eco-centric concern (EC)*	0.82
*Scale 12*: *Support for population growth policies (SP)*	0.85

### Results

All data analyses were performed using SPSS version 18.0 [59]. The unidimensionality of the EAI was checked, and a principal component analysis (PCA) using the extraction method of principal axis factoring with oblique rotation was performed. The EAI factors were proven to be statistically important according to the Kaiser-Meyer-Olkin measure of sampling adequacy of 0.891 and Bartlett's test of Sphericity (χ2 = 4458.963, df = 666, p<0.000); this indicates that the non-existence of multicollinearity and correlations between items were sufficient to perform a factor analysis. The eigenvalues for each scale were more than 1.0, and the cumulative percentage variance as 75.564. The following scales were excluded from the EAI dimensions: Scale 1-Enjoyment of nature, Scale 6-Environmental fragility, Scale 8-Personal conservation behavior, Scale 9-Human dominance over nature, Scale 12-Support for population growth policies. The reduction of items was due to cross-loadings and factor loading values of less than 0.5 as suggested by Hair et al. [60]. The EFA was performed using varimax rotation to examine the dimensionality of the items on the 7-factor constructs of the 35 items. The EFA demonstrated that the 35-item EAI was rotated into 7-factor models of the EAI (Table 3) without losing information and internal consistency. The data were considered normal because skewness and kurtosis were between the acceptable limits of ±2.0 [61] and ±7.0, respectively [62].

**Table 3:** Results of the factor analysis of the EAI scales (35 items) (Study 1).

Dimensions/Items		Factor loadings	Eigenvalues	% of Variance
Scale 2-Support for interventionist conservation policies (SI)			15.367	41.532
	SI4	0.907 (0.881)		
	SI5	0.861 (0.802)		
	SI6	0.846 (0.810)		
	SI2	0.823 (0.859)		
	SI3	0.807 (0.839)		
	SI1	0.799 (0.835)		
	CST6	0.707 (0.691)		
	CST5	0.582 (0.580)		
Scale 11-Eco-centric concern (EC)			3.891	10.517
	EC4	0.857 (0.737)		
	EC3	0.840 (0.835)		
	EC5	0.811 (0.706)		
	EC1	0.765 (0.846)		
	EC6	0.751 (0.667)		
	EC2	0.749 (0.845)		
Scale 3-Environmental movement activism (EM)			3.078	8.320
	EM3	0.835 (0.806)		
	EM1	0.825 (0.820)		
	EM4	0.805 (0.781)		
	EM5	0.790 (0.733)		
	EM2	0.784 (0.889)		
	EM6	0.755 (0.679)		
Scale 4-Conservation motivated by anthropocentric concern (CM)			1.929	5.214
	CM2	0.719 (0.890)		
	CM3	0.705 (0.778)		
	CM1	0.694 (0.882)		
	CM4	0.673 (0.587)		
Scale 10-Human utilization of nature (HU)			1.395	3.771
	HU2	0.819 (0.881)		
	HU3	0.717 (0.894)		
	HU1	0.717 (0.820)		
Scale 7-Altering nature (AN)			1.204	3.254
	AN3	0.599 (0.541)		
	AN4	0.578 (0.620)		
	AN6	0.575 (0.783)		
	AN2	0.552 (0.670)		
	AN1	0.545 (0.601)		
Scale 5-Confidence in science and technology (CST)			1.094	2.956
	CST2	0.749 (0.923)		
	CST3	0.704 (0.764)		
	CST1	0.673 (0.784)		

Note: Factor loadings of the CFA are in parentheses.

To examine the adequacy of convergent and discriminant validity, a confirmatory factor analysis (CFA) was performed using SPSS AMOS version 23.0. The measurement model of Study 1 produced an adequate convergent and discriminant validity, met the threshold suggested for factor loadings (≥0.5) [63], AVEs (>0.5) [63], the Cronbach's Alpha (CA) (>0.60) [64], and composite reliability (CR) (≥0.60) [64] (Table 4). A cut-off value of less than 0.80 for RMSEA [62], 0.80 for the NFI [65], and 0.90 for the TLI and CFI [66] were necessary due to the sensitivity to some variables and insensitivity to the sample size. Thus, it indicated acceptable goodness-of-fit indices (CMIN = 899.236; CMIN/df = 1.716; CFI = 0.907; NFI = 0.805, TLI = 0.894 and RMSEA = 0.074).

**Table 4:** Results of the Bivariate correlation analysis of the 7 dimensions (35 items) (Study 1).

		1	2	3	4	5	6	7
1	Scale 2-Support for interventionist conservation policies (SI)	1						
2	Scale 11-Eco-centric concern (EC)	0.488** (0.*238)*	1					
3	Scale 3-Environmental movement activism (EM)	0.339** (0.*159)*	0.438** (0.*192)*	1				
4	Scale 4-Conservation motivated by anthropocentric concern (CM)	0.605** (0.*366)*	0.612** (0.*375)*	0.548** (0.*300)*	1			
5	Scale 10-Human utilization of nature (HU)	0.455** (0.*207)*	0.628** (0.*394)*	0.384** (0.*147)*	0.465** (0.*216)*	1		
6	Scale 7-Altering nature (AN)	0.591** (0.*349)*	0.613** (0.*376)*	0.510** (0.*260)*	0.507** (0.*257)*	0.599** (0.*359)*	1	
7	Scale 5-Confidence in science and technology (CST)	0.129 *(0*.*017)*	0.228** (0.*052)*	0.371** (0.*138)*	0.220* (0.*048)*	0.198* (0.*039)*	0.258** (0.*067)*	1
	Mean	5.30	5.62	6.03	5.61	5.61	5.62	5.57
	*SD*	1.037	0.741	0.616	0.894	0.795	0.637	0.318
	Skewness	-1.213	-0.605	-0.364	-1.255	-0.419	-0.353	1.342
	Kurtosis	2.270	0.580	0.397	2.511	0.198	0.499	2.737
	AVE	0.636	0.635	0.639	0.487	0.566	0.325	0.503
	CA	0.792	0.796	0.799	0.698	0.751	0.570	0.709
	CR	0.932	0.912	0.914	0.792	0.796	0.706	0.752

** Correlation is significant at the 0.01 level (2-tailed)

* Correlation is significant at the 0.05 level (1-tailed); *(r*^*2*^*)*

## Study 2

One step towards establishing validity and reliability is the use of the repeatability approach, in which measurements are done under the same conditions and within a short period of time using the same 72-item 12-dimension EAI instrument [67]. This method was used to test the stability and reliability of the 35 items of the 7-dimension EAI (confirmed in Study 1) over time. AMOS version 23.0 [68] was used to test Anderson and Gerbing's [69] two-step modeling method to examine the measurement model. A CFA was performed for the individual construct and measurement model.

### Participants and Instruments

A month after conducting Study 1, 400 anonymous and consented questionnaires were administered to the same sample of final year undergraduates at Abu Dhabi University, UAE. Only 32.5% (130) of the subjects were qualified for the study based on the criterion of giving complete and consistent feedback. The non-response rate was 65.0% (260), and five incomplete responses were withdrawn. The withdrawn responses had similar socio-demographic profiles (males, and higher age group). The participants with valid responses were 83 females and 47 males, ranging in age from 21 to 35 years (M = 25, SD = 0.785), and social sciences and science and technology college students. Study 2 adopted the procedure of Study 1 to test Milfont and Duckitt’s [22] EAI items.

### Results

**Fig 1:**
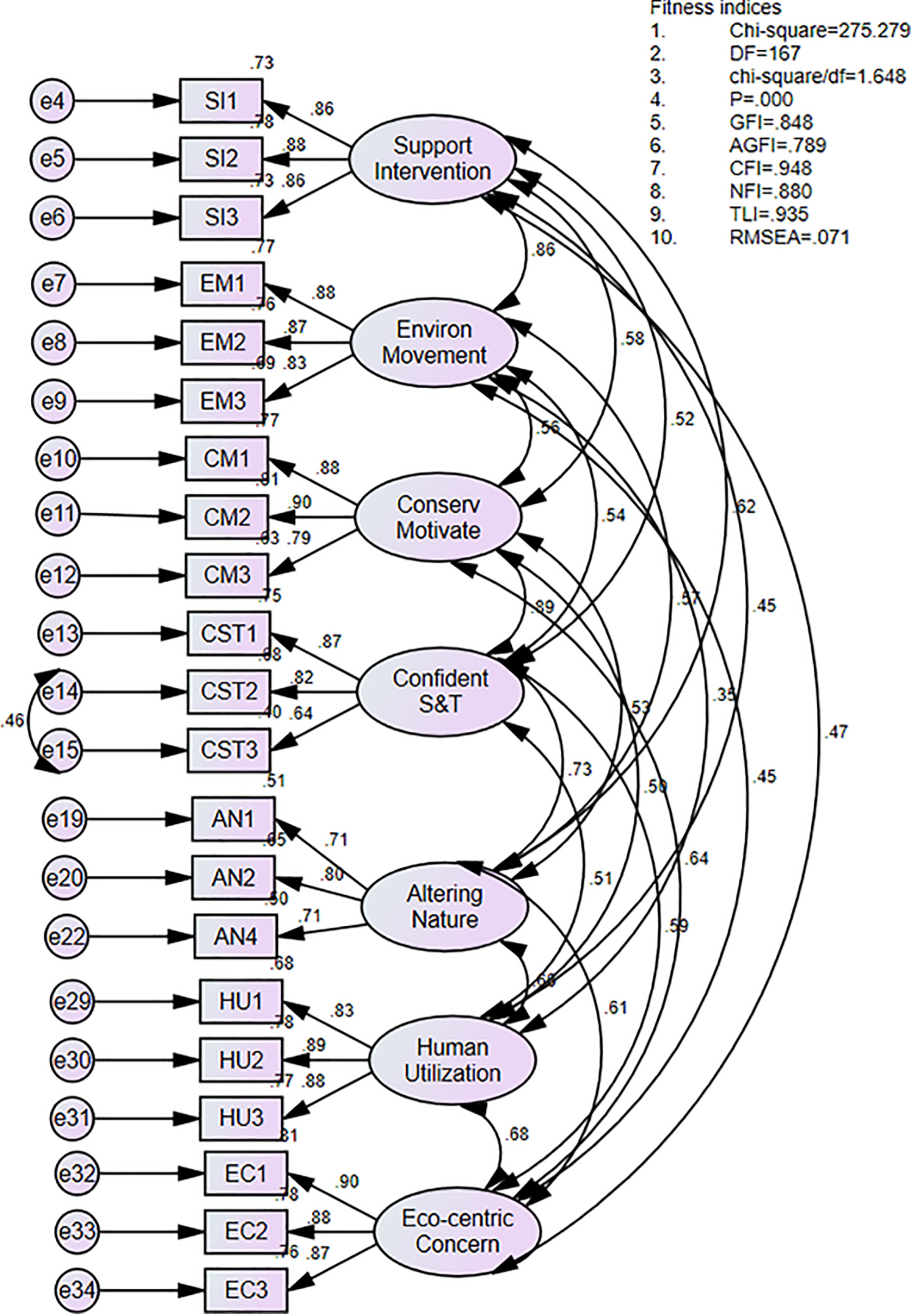
Final structural model of the 7-factor model; 21 items.

A CFA was performed on the 72 items of the 12-dimension EAI and measurement model; the final model confirmed 21 items of the 7-factor model of the EAI (Table 5). The acceptable limits for skewness and kurtosis were ±3.0, corresponding to a normal distribution of the data [70]. The factor loadings (Table 6) and AVEs were greater than 0.50 [71]. The CRs and CAs were well above 0.70, suggesting good convergent validity of the studied variables [62]. All seven constructs were between 0.347 (r^2^ = 0.120) and 0.858 (r^2^ = 0.736) at p<0.01, with AVEs greater than r^2^, exhibiting sufficient discriminant validity) (Table 7). In Study 2, all measurement models yielded satisfactory goodness-of-fit. However, five constructs, Scale 1-Enjoyment of nature, Scale 6-Environmental fragility, Scale 8-Personal conservation behavior, Scale 9-Human dominance over nature, and Scale 12-Support for population growth, were withdrawn because the r^2^ values were higher than AVEs, thereby indicating insufficient discriminant validity among the constructs [62]. As a result, the number of items was reduced from 72 to 21 for the 7-factor model. The final 7-factor model of the EAI yielded satisfactory goodness-of-fit indices (CMIN/df = 1.648; CFI = 0.948; NFI = 0.808, TLI = 0.935 and RMSEA = 0.071). The final structural model of the 7-factor model is illustrated in Fig 1. A cut-off of less than 0.80 for RMSEA [62], 0.80 for the NFI [65] and 0.90 for the TLI and CFI [66] are necessary due to the sensitivity to some variables and insensitivity to the sample size. A CFI greater than 0.90 indicates adequate fit [72] and can be reproduced for future study.

**Table 5:** Fit indices for alternative models (Study 2).

Model/s	Variable	# items	χ^2^	χ^2^/*df*	GFI	CFI	NFI	TLI	RMSEA
1 factor	EN	3							
2 factors	EN;SI	3;3	4.162	0.520	0.990	1.000	0.990	1.000	0.000
3 factors	EN;SI;EM	2;3;3	26.995	1.588	0.953	0.986	0.964	0.977	0.068
4 factors	EN;SI;EM;CM	2;3;3;3	55.303	1.495	0.934	0.982	0.949	0.973	0.062
5 factors	EN;SI;EM;CM;CST	2;3;3;3;3	105.018	1.591	0.904	0.971	0.926	0.960	0.068
6 factors	EN;SI;EM;CM;CST;EF	2;3;3;3;3;3	186.231	1.808	0.867	0.954	0.904	0.939	0.079
7 factors	EN;SI;EM; CM;CST;EF; AN	2;3;3;3;3;3;4	291.879	1.748	0.836	0.940	0.872	0.924	0.076
8 factors	EN;SI;EM;CM;CST;EF;AN;PC	2;3;3;3;3;3;4;3	353.283	1.758	0.823	0.939	0.871	0.923	0.077
9 factors	EN;SI;EM;CM;CST;EF;AN;PC;HD	2;3;3;3;3;3;4;3;3	466.782	1.775	0.798	0.927	0.850	0.910	0.078
10 factors	EN;SI;EM;CM;CST;EF;AN;PC;HD;HU	2;3;3;3;3;3;4;3;3;3	598.436	1.803	0.744	0.916	0.833	0.897	0.079
11 factors	EN;SI;EM;CM;CST;EF;AN;PC;HD;HU;EC	2;3;3;3;3;3;4;3;3;3;3	728.611	1.786	0.761	0.912	0.824	0.893	0.078
12 factors	EN;SI;EM;CM;CST;EF;AN;PC;HD;HU;EC;SP	2;3;3;3;3;3;4;3;3;3;3;3	887.528	1.804	0.745	0.906	0.815	0.886	0.079
10 factors **	SI;EM;CM;CST;EF;AN;HD;HU;EC;SP	3;3;3;3;3;4;3;3;3;3	639.826	1.797	0.778	0.920	0.839	0.902	0.079
8 factors **	SI;EM;CM;CST;AN;HU;EC;SP	3;3;3;3;4;3;3;3	386.454	1.733	0.818	0.937	0.866	0.923	0.075
7 factors **	SI;EM;CM;CST;AN; HU;EC	3;3;3;3;3;3;3	275.279	1.648	0.848	0.948	0.880	0.935	0.071

** Revised model–constructs withdrawn due to correlation is not significant at the 0.05 level.

**Table 6:** Factor loadings of the 21 items of the 7 dimensions (Study 2).

Dimensions/Items		Factor loadings
Scale 2-Support for interventionist conservation policies (SI)		
	SI1	0.86
	SI2	0.88
	SI3	0.86
Scale 11-Eco-centric concern (EC)		
	EC1	0.90
	EC2	0.88
	EC3	0.87
Scale 3-Environmental movement activism (EM)		
	EM1	0.88
	EM2	0.87
	EM3	0.83
Scale 4-Conservation motivated by anthropocentric concern (CM)		
	CM1	0.88
	CM2	0.90
	CM2	0.79
Scale 10-Human utilization of nature (HU)		
	HU1	0.83
	HU2	0.89
	HU3	0.88
Scale 7-Altering nature (AN)		
	AN1	0.71
	AN2	0.80
	AN3	0.71
Scale 5-Confidence in science and technology (CST)		
	CST1	0.87
	CST2	0.82
	CST3	0.64

**Table 7:** Results of the Bivariate correlation analysis of the 21 items of the 7 dimensions (Study 2).

		1	2	3	4	5	6	7
1	Scale 2-Support for interventionist conservation policies (SI)	1						
2	Scale 3-Environmental movement activism (EM)	0.858** (0.*736)*	1					
3	Scale 4-Conservation motivated by anthropocentric concern (CM)	0.576** (0.*332)*	0.559** (0.*312)*	1				
4	Scale 5-Confidence in science and technology (CST)	0.463** (0.*214)*	0.477** (0.*228)*	0.839** (0.*704)*	1			
5	Scale 7-Altering nature (AN)	0.620** (0.*384)*	0.566** (0.*320)*	0.529** (0.*280)*	0.711** (0.*506)*	1		
6	Scale 10-Human utilization of nature (HU)	0.452** (0.*204)*	0.347** (0.*120)*	0.497** (0.*247)*	0.509** (0.*259)*	0.659** (0.*434)*	1	
7	Scale 11-Eco-centric concern (EC)	0.468** (0.*219)*	0.454** (0.*206)*	0.639** (0.*408)*	0.551** (0.*304)*	0.613** (0.*376)*	0.677** (0.*458)*	1
	Skewness	-0.488 - -0.620	-0.401 -0.661	-0.857 - - 1.052	-0.003 - -1.091	-0.552 - -0.685	-0.203 - -0.893	-0.550 - -0.747
	Kurtosis	0.261 0.452	0.279 -0.576	0.808 -1.354	1.206 -2.015	0.509 -1.876	0.148 -1.627	0.540 -1.042
	AVE	0.746	0.740	0.736	0.671	0.555	0.746	0.780
	CA	0.863	0.860	0.857	0.817	0.743	0.863	0.883
	CR	0.898	0.895	0.893	0.859	0.788	0.898	0.914

** Correlation is significant at the 0.01 level (2-tailed)

* Correlation is significant at the 0.05 level (1-tailed); *(r*^*2*^*)*

## Discussion

This paper reports on the findings of two studies that investigated whether the 12-factor model identified in previous validation studies is suitable for a new dataset from student subjects [22, 34, 47]. Both studies were conducted on the same target sample with a different timeline to understand the extent of environmental attitudes using the test-retest approach. These approaches developed different models to arrive at an appropriate instrument for use in future research. The EFA and CFA were conducted in Study 1, and the model fits were discussed. Study 1 examined the EAI unidimensionality and dimensionality of each construct following the ABC of test construction [67]. EAI as the construct being measured is clearly discussed. The test-retest analysis was used to justify the intended use of the EAI measure and its use among undergraduate students in the Arab context. This development strategy has provided the basis for the correct use of the test for future study and can be used by other researchers when building on these findings. In Study 2, the CFA was conducted to evaluate parameter estimates, fit indices, and potential modification indices to facilitate theory-testing. This is consistent with the suggestion of earlier studies, which concur that psychometric analysis is applicable for construction and validation of assessment instruments concerned with attitudes [6, 8, 11, 28, 42–43]. The CFA was performed to test the new factor structure from the dataset and compare it with the 7-factor model in Study 1. The data were obtained from educated subjects, consistent with earlier environmental attitude-related studies, which posited that a well-educated sample was expected to be more exposed to information about ecological issues and be more capable of understanding the ecological perspective implied in the measurement [24, 34].

In Study 1, the EFA was conducted to extract the new factor structure of the dataset and identified the 7-factor structural model (Table 3). The 35 items of the EAI were shown to have better unidimensionality, which indicated that the non-existence of multicollinearity and correlations between items was sufficient for a factor analysis of the 7-factor model. The eigenvalues for each scale were more than 1.0, and the cumulative percentage variance was 75.564. These findings are consistent with earlier studies, which concur that the EAI shows evidence of unidimensionality [8, 22, 47]. The unidimensionality was shown in this study because the items of the 7-factor model with 35 items had greater loadings on their corresponding factors and almost all items could be explained by one factor. The unidimensionality of each dimension of the EAI was further examined in the EFA for each factor using a test-retest approach to ensure balanced models. The criteria for convergent validity and discriminant validity of the measurement model and item balance were also confirmed (Table 3). In comparison with the previous studies, this study’s dataset had 7 factors that were more well-separated [39, 42–44].

In general, factor loadings should be equal to or greater than 0.50 for good convergent validity [63]. All 35 items had factor loadings greater than 0.50, indicating high convergent validity. The CA and CR values met the threshold value of at least 0.70 per dimension [64]. All 7 factors of the model had high convergent validity except for Scale 7-Altering nature (AN) (CA = 0.57). Thus, these models may influence the independence of the final structural model. The low convergent validity of Scale 7-Altering nature (AN) was due to low factor loadings of all 5 items; thus, items from this factor may overlap with other factors [73]. Cho [74] argues that CA tends to systematically underestimate reliability due to the assumption that all factor loadings are equal. Other scholars have posited that CR is a reliable measure of reliability [64, 74]. These recommendations support the acceptance of Scale 7-Altering nature (AN) into the construct (CR = 0.71). In this study, the average variance extracted (AVE) for the 5-factor model was satisfactory, meeting the threshold of 0.5 [71], except for Scale 4-Conservation motivated by anthropocentric concern (CM) (AVE = 0.49) and Scale 7-Altering nature (AN) (AVE = 0.33). Fornell and Larcker [71] have suggested that CR must be higher than 0.6 if the AVE is less than 0.5, to support the acceptance of a factor model.

Bivariate-correlation (r) values for each scale were significant with p<0.05, between 0.198 and 0.628 and r^2^ ranging from 0.052 to 0.394. AVEs also were found to have a value greater than the r^2^ values, indicating sufficient discriminant validity [75–76]. The shortened version of the 35 items was also found to be similar to that of Milford and Duckitt [22] in that it had a mixture of positive and negative worded items in each scale to prevent response bias. This result indicates that the 7-factor model with 35 items of the EAI can be causally interpreted [66]. These test constructions align with earlier studies [72] that have suggested that a construct should be measured for unidimensionality before a series of model tests for multidimensionality can be conducted. The unidimensionality of a construct is important as the nomological items should be defined and interpreted before results can be obtained regarding the structural model and ideally replicated. Therefore, in this model and dataset, the factors were associated with one another. There are two possible explanations. First, the latent factors constituting the environmental attitudes cannot be independent. Additionally, since perceptions of the consequences of an individual’s actions may reflect their responsibilities, the factors are closely associated and expressed together. Second, since the subjects were highly educated, it is possible that they were more exposed to ecological issues due to the abundance of information provided to them. As a result, they could relate the value of ecology to concerns about ecology.

Study 1 considered the criteria for various model fit indices to discuss the model fit of the CFA. It has been suggested that RMSEA values less than 0.08 are acceptable [77]. Therefore, the RMSEA value of 0.074 in this sample indicates an acceptable fit. In this sample, the CFI value of 0.907, NFI value of 0.805, and TLI value of 0.894 are considered an acceptable fit. Even though it has been argued that the NFI and TLI values should be over 0.90 to indicate good fit, a value between 0.80 and 0.90 (0.80<NFI<0.90) is generally considered an acceptable limit [62–63, 75, 78]. Based on these indices, this sample had an acceptable fit for the 7-factor model. It has provided a measure for the assessment of the EAI in a specific cultural context, and for reproduction in future studies. Furthermore, this illustrates ways to assess the usefulness of a model in a new setting.

Although the EAI was shown to be the new culture-general and fully-balanced tool for measuring environmental attitudes [34, 47], the validity and test-retest reliability in the Arab context has yet to be measured. In this study, the factor structure of the EAI was investigated by both EFA and CFA in both studies based on the suggestion of Altman and Bland [79] that repeatability will evaluate the accuracy of responses. Thus, applying the CFA as a step forward to the EFA to test the EAI is reasonable for this study because it measures the stability and reliability of the original instrument in a different context using the same sample size, repeated over a short period of time, using the same 72-item 12-dimension EAI. Study 2 is a step forward for EAI testing. Study 2 tested alternative factor models to identify the best fitting factor model that sufficiently met the goodness-of-fit indices thresholds to explain the EAI better. Even though the EAI was tested in multiple countries across continents using undergraduate students and was found to have reasonable internal consistency and homogeneity across samples, this study showed a different outcome. Fifteen models were tested, and fit indices were generated. Of these 15 models, the 7-factor model (21 items) showed a better model fit (CMIN/df = 1.648; CFI = 0.948; NFI = 0.808, TLI = 0.935, and RMSEA = 0.071) (Table 4). The rest of the constructs were withdrawn due to factors being insignificant (p>0.05). The 7-factor model with 21 items showed an improved model fit in comparison to Study 1 (CMIN/df = 1.716; CFI = 0.907; NFI = 0.805, TLI = 0.894, and RMSEA = 0.074). This result indicated that the 21-item EAI is a reliable and valid tool for investigating multidimensional environmental attitudes due to its sufficient convergent validity and discriminant validity to support the development of the measurement model (Table 6). In line with previous studies of NEP/R [21, 23] and EAI [22], psychometric testing yielded different numbers of dimensions—between two and five for NEP/R [26, 27, 28] and between six and eleven for the EAI short version [34, 39, 42, 44]. Even though studies have reported that the 12-factor model offers empirical evidence [22, 39], the findings of these studies showed an insignificant correlation value in the 12-factor model; however, dimensions were not withdrawn. The factor that was not withdrawn due to its insignificance led to misleading results because the coefficient of correlation measures the strength associated with the dimensions and determines how much the variables have been changed due to insufficient discriminant validity between the dimensions (AVEs < r^2^).

These reported results support the validity and reliability of Milford and Duckitt's [22] EAI. Overall, the 7-factor model was shown to have unidimensional factors with high internal consistency, homogeneity, and test-retest reliability; however, it is not free from cultural bias. This is consistent with earlier studies, which showed that socio-demographic factors affect social-desirability bias and the internal consistency of the data of environmental attitudes studies [21, 45–46]. The replication of the EAI version in the Arab context in Study 1 and Study 2 was shown to extract factor structures that were different from the original constructs. It was found that Scale 1-Enjoyment of nature (EN), Scale 6-Environmental fragility (EF), Scale 8-Personal conservation behavior (PC), Scale 9-Human dominance over nature (HD), and Scale 12-Support for population growth policies (SP) were not considered and perceived necessary by students in the Arab context. Differences in the factor structures affected by the psychological constructs of the respondents of the current study and previous studies may be due to the cultural context that contributed to social-desirability bias.

This is supported by scholars and previous studies who stated that dismissing this factor may result in a statistical bias [22, 34, 42–44]. Wu [80] argued that results from previous research were not adequate for diverse populations and not sustained across cultures. Even though the characteristics and practices of the target population are common, cultural differences exist between norms, beliefs, and values; this may represent the social-desirability bias in the current study. Study 1 and Study 2 have established a baseline framework for environmental attitudes in the Arab context. The shortened version of the EAI was found to be reliable, valid, and sufficiently detailed in all aspects of environmental traits. In both studies, more than 40% of the sample did not provide usable data; this may have led to non-response bias affecting the results of the current study. Even though Howcroft and Milfont [37] have criticized the utility of the NEP/R for comparing scores across studies with different sample types and scale lengths, the usability of the EAI was found to be challenging due to a large number of items and redundancy in the positive and negative items. Feedback received from participants suggested that many students found the questionnaire to be excessively long and many of the items overly repetitive. The long-winded questionnaire of the original EAI has contributed to fatigue and a high percentage of unusable data.

Despite acknowledging this feedback, this study maintained the 72 items of the EAI to measure its dimensionality, reliability, and validity and to establish the utility of the environmental attitudes measure in the Arab context due to its psychometric stability across cultures [34, 47]. Since no study has been conducted in the Arab context to measure the reliability and validity of an environmental attitudes tool for investigating multidimensional environmental attitudes, it was rational and practical to adopt the EAI construct for the current study. The results from Study 1 and Study 2 revealed that the extraction of the items into a 7-factor model from the 12-factor model with between three and eight items for each dimension in Study 1, and three-item scales of all seven dimensions in Study 2 did not compromise the scale's internal consistency and reliability; thus, the content validity of the environmental attitude is intact.

Furthermore, the test-retest method is known for providing the opportunity for respondents to recall their feedback that would most likely impact their subsequent responses. Even though Altman and Bland [79] have suggested that certain criteria such as the same sample, same location, and a short interval between the tests must be fulfilled when establishing the repeatability approach, the test-retest approach entails the risk of recall bias due to the short time gap between the first and second studies [81]. Study 2 showed a significant reduction in the amount of complete and consistent feedback at 32.5% in comparison to Study 1 with 52.0%. A long-winded questionnaire and fatigue might have contributed to the signification reduction in the number of responses in these two studies. Since the responses from both studies were retrieved from the same sample, the negative experiences from the first study may have influenced the undergraduate students to provide void responses [82].

The usage of EFA should be strictly considered due to its usability as a data-driven method [76, 81]. On the other hand, CFA should be chosen to balance the aims of the study and concerns on theory-driven method [69, 76]. Even though some argue that EFA can explore the latent variable structure of a dataset [83], Byrne [57] contended that the CFA is the most robust method as it requires an a priori hypothesis to test whether the obtained dataset is suitable for the model. In this study, the PCA and EFA in Study 1 were used to verify the unidimensionality of the construct and were followed by the examination of a new factor structure extraction. The method was test-retesting, allowing for CFA to measure the model fit of the 7-factor model obtained from the same dataset in a different timeline. Unlike earlier studies that measured cross-cultural EAI on an educated sample, this study offers a different approach to administering the test on a well-educated sample, given the aim of determining the suitability of the EAI in the Arab context to establish the baseline framework. This may have resulted in a small difference in the factor structure. Despite confirmation of a similar structure in both studies, the relatively low reliability and validity of a few factors in Study 1 may also have contributed to the relative difference between factor structures.

Consistent with earlier studies, this study considered undergraduate students as educated (e.g.; [10, 34, 36, 38, 39, 43, 71] because they have learned about and experienced sustainability through curriculum, shared practice, and collaborative learning via co-curricular activities, experiences through sustainability research, and interdisciplinary collaboration [83]. Prior research has also proven that undergraduate students have a reasonable level of environmental awareness [10, 36, 34] to justify their attitudes toward pro-environmental behavior.

To overcome these limitations, and to acquire predictability and validity, prospective studies should include the gold standard of socio-demographic factors to further compare the environmental attitudes toward ecology issues. Zsoka et al. [84] clarified that previous studies have focused on the importance of attitude modeling. Sutton and Gyuris [47] have urged for future studies to explicitly address the need to measure change in people’s affective environmental attributes or actual behavior as a consequence of their ecological experiences. Although many studies have associated environmental attitudes with education and measures for a significant duration of the university experience, Chen et al. [85] argued that the effort to study environmental attitudes could be extended to less educated people, those with low employment status, and inhabitants of smaller cities. The uncommon function of the psychosocial aspect in environmental psychology studies in measuring environmental attitudes is consistent with suggestions that the social cost of environmental attitudes varies by social and cultural contexts. Thus, educational level may be helpful but not necessarily sufficient [85–89].

## Conclusions

Collectively, this study provides a baseline for the environmental attitudes inventory in the Arab context. The results indicate that the 21-item scale of the EAI in Study 2 is a reliable and valid tool for investigating multidimensional environmental attitudes. Potential response bias due to response burden can be reduced. A reduction in some items of the scale will improve the response rate and completion rate compared to the longer version of the EAI. Furthermore, potential pitfalls due to cultural bias and social-desirability bias can be controlled for and minimized because items that are not considered important in relation to the environmental issues can be excluded from the survey. This paper established that the 72-item EAI with 12 dimensions by Milford and Duckitt [22] is a potential gold standard to measure environmental attitudes in the Arab context. It can be presented as a 21-item instrument to reduce the response burden, increase the response rate, and improve the completion rate while preserving the balance, dimensionality, reliability, and validity of the original version.
